# Emergence of *Pseudomonas aeruginosa* cross-infection in children with cystic fibrosis attending an Iranian referral pediatric center

**Published:** 2012-09

**Authors:** M Ghazi, G Khanbabaee, F Fallah, B Kazemi, S Mahmoudi, M Navidnia, B Pourakbari, Bakhshi B, H Goudarzi

**Affiliations:** 1 Department of Microbiology, School of Medicine, Shahid Beheshti University of Medical Sciences, Tehran, Iran; 2 Department of Pediatric Respiratory Diseases, Mofid Children Hospital, Shahid Beheshti University of Medical Sciences, Tehran, Iran; 3 Pediatric Infectious Research Center, Shahid Beheshti University of Medical Sciences, Tehran, Iran; 4 Cellular and Molecular Biology Research Center, Shaheed Beheshti University of Medical Sciences, Tehran, Iran; 5 Pediatric Infectious Disease Research Center, Tehran University of Medical Sciences, Tehran, Iran; 6 Department of Bacteriology, Tarbiat Modares University, Tehran, Iran

**Keywords:** *P. aeruginosa*, Cystic fibrosis, genotyping, Iran

## Abstract

**Background and Objectives:**

This study was carried out with the objective of determining the genomic variability of *P. aeruginosa* strains isolated from patients suffering from cystic fibrosis or from environmental cultures collected from different locations in the unit they admitted.

**Materials and Methods:**

A total of 57 clinical and environmental *P. aeruginosa* isolates were genotyped by enterobacterial repetitive intergenic consensus-PCR (ERIC-PCR), and antimicrobial susceptibility testing was performed using the Clinical and Laboratory Standards Institute method.

**Results:**

One predominant ERIC profile (type A) was identified in 46 strains (81% of all typed isolates) which was responsible for thirty-nine of 44 clinical isolates (89%) and 7 of 13 environmental isolates (54%). All clinical isolates were susceptible to piperacillin-tazobactam, ceftazidime and cefepime followed by ticarcillin, aztreonam, amikacin and tobramycin (96.5%).

**Conclusions:**

In our country CF patients are not segregated from other patients, and transmission of bacteria between these patients and other patients might occur in the wards via personal contact or contaminated environment. Future evaluation for policy of patient segregation is necessary and the elimination of contaminated sources and control of environmental spread and recurrent contamination risk is needed.

## INTRODUCTION

Cystic fibrosis (CF) is the most common lethal genetic disease in the white population and its prevalence is 1 case per 90,000 in Asia (1). *Pseudomonas aeruginosa* is the major opportunistic pathogen in this disease (2). If this disorder is intensively treated, the mean expected life expectancy of CF patients will be >35 years and up to even 50 years in some centers, otherwise most die at a young age (3).

The emergence of epidemic *P. aeruginosa* strains that infect CF patients has significant implications for infection control within the hospital (4). Several molecular methods with high reproducibility and good discriminatory power have been developed to study *P. aeruginosa* strains isolated from the sputa of patients with CF (5). Generally, the discriminatory powers of the typing methods, including enterobacterial repetitive intergenic consensus PCR (ERIC-PCR) and pulsed-field gel electrophoresis (PFGE), were equivalent and strong correlation between the information provided by these typing methods (6).

This work was carried out with the objective of determining the genomic variability of *P. aeruginosa* strains isolated from patients suffering from cystic fibrosis or from environmental cultures collected from different locations in the unit they admittedby ERIC-PCR.

## MATERIALS AND METHODS

### Bacterial isolates

Twenty-three CF patients who were admitted to the Mofid's Children Hospital (a tertiary referral pediatrics center affiliated to Shahid Beheshti University of Medical Sciences) because of a pulmonary exacerbation or acute complications associated with the chronic airways infection were included in the study. All of these patients were hospitalized in respiratory ward.

*P. aeruginosa* isolates were collected from different sources by the Microbiology Department of this Hospital. In a number of cases, multiple isolates from a single patient over different periods of time at least one week after antibiotic treatment were collected and identified as separate isolates in this study. *P. aeruginosa* isolates were recovered by bacterial isolation on MacConkey agar plates and identified by colony size, pigmentation and mucoidy, Gram staining, growth at 42°C, biochemical tests including carbohydrate fermentation of glucose, lactose and sucrose, lysine decarboxylase, ornithine decarboxylase, indol, citrate assimilation, and oxidase (7).

Environmental cultures were collected from locations in the unit that CF patients admitted, including patient rooms and communal areas using a premoistened swab. Particular attention was paid to moist areas such as sinks, taps and soap dispensers, where *P. aeruginosa* is known to proliferate. Door handles and objects in communal areas such as the refrigerator were also sampled.

### Antimicrobial susceptibility testing

The following antibiotics were tested: piperacillin-tazobactam (110 µg), ticarcillin (75 µg), ceftazidime (30 µg), cefepime (30 µg), aztreonam (30 µg), gentamicin (10 µg), amikacin (30 µg), tobramycin (10 µg), ciprofloxacin (5 µg), imipenem (10 µg), meropenem (10 µg), ertapenem (10 µg), doripenem (10 µg) and trimethoprim-sulfamethoxazole (5 µg). The antimicrobial susceptibility testing was carried out through the disk diffusion method according to CLSI recommendation (8).

### Extraction of bacterial DNA

DNA was extracted using the QIAamp DNA Mini Kit (QIAGEN), following the manufacturer's instructions. Purified nucleic acid was eluted from the extraction column in 50 µl elution buffer and stored at -70 ^◦^C until analysis.

### ERIC-PCR

ERIC-PCR typing of the clinical and environmental isolates using two oligonucleotide primers, ERIC1 (5’ATGTAAGCTCCTGGGGATTCAC3’) and ERIC2 (5’ AAGTAAGTGACTGGGGTGAGCG 3’) was done as described previously (9). PCR amplification was performed using an initial denaturation step at 94°C for 7 min, followed by 30 cycles of denaturation at 94°C for 1 min, annealing at 53°C for 1 min, extension at 72°C for 2 min and one cycle of further extension at 72°C for 15 min. Comparison of ERIC-PCR banding patterns was performed using Gelcompar II software, version 6.5 (Applied Maths, Sint-Matens-latem, Belgium). Each gel photograph was inverted as TIFF images and then normalized by using the reference marker. Similarity analysis of results was calculated using the Dice coefficient/ unweighted pair-group method with arithmetic mean (UPGMA). The criterion for related clones was taken as profiles with 80 %or more similar bands. Isolates with 100% similarity were considered as the same ERIC-PCR type.

## REULTS

All patients were between 4 months to 15 years of age at enrollment and had a confirmed diagnosis of CF by sweat test. The average ages of the studied patients were 7.4 years and the majority of patients (56%) were male. A total of 57 clinical and environmental *P. aeruginosa* isolates were genotyped by ERIC-PCR (44 clinical and 13 environmental isolates).

**Fig. 1:**
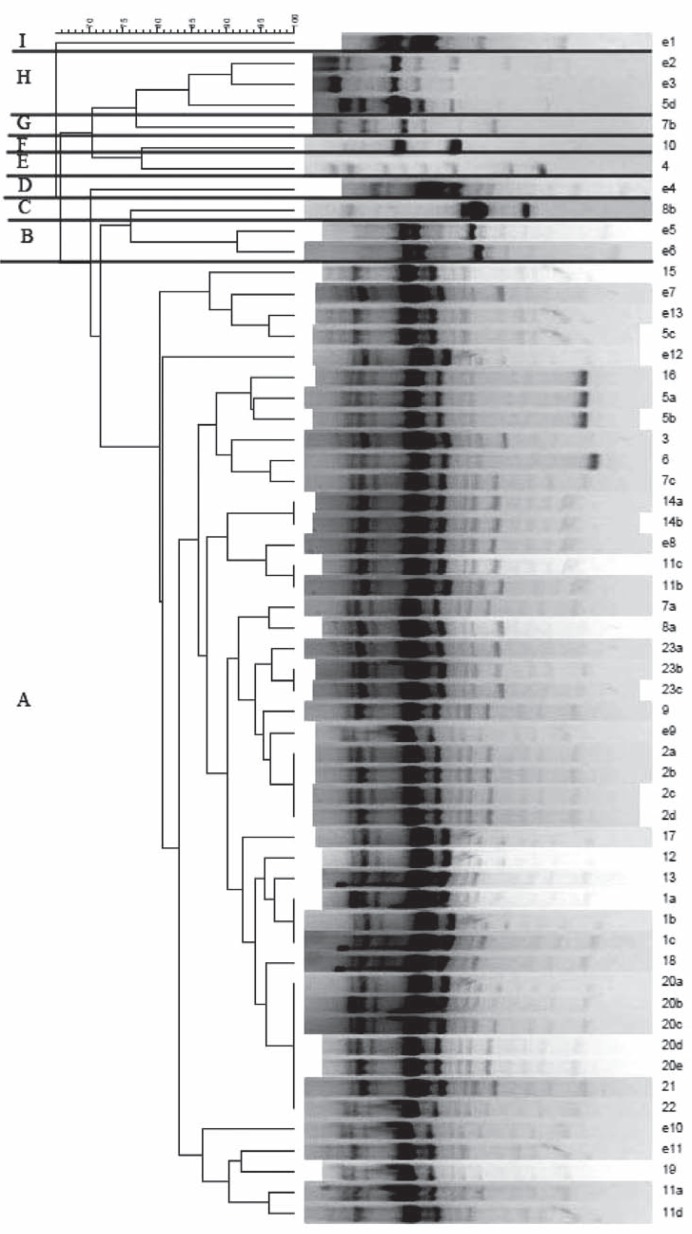
Dendrogram of ERIC-PCR results for all *P. aeruginosa* isolates. The scale at the top represents the genetic distance between the isolates.

One predominant ERIC profile (type A) was identified in 46 strains (81% of all typed isolates) which was responsible for thirty nine of 44 clinical isolates (89%) and 7 of 13 environmental isolates (54%). Three patients displayed an A profile and other different patterns (C, G, H). Only 2 patients showed different profiles with genetic diversity that was not found in other patients (profile E and F) (Fig. 1).

Isolates of 8 patients showed identical pattern (all isolates of case 1, 2, 14, 20, 21, 22 and two isolates of case 11 and 23) (Table 1). It was possible to obtain more than one isolate from the 9 patients over a period of time of up to 1 year and most of these patients tended to harbor a single strain (genotype) over many months.

**Table 1:** Age and sex distribution of CF patients and types of *P. aeruginosa* isolated from them.

Case	Age (year)	Sex	Number of isolates	Isolated type
1	15	M	3	A, A, A
2	12	M	4	A, A, A, A
3	14	F	1	A
4	8	F	1	E
5	13	M	4	A, A, A, H
6	10	M	1	A
7	1	M	3	A, A, G
8	2	F	2	A, C
9	8	M	1	A
10	0.6	F	1	F
11	11	F	4	A, A, A, A
12	10	F	1	A
13	14	F	1	A
14	1	F	2	A, A
15	14	F	1	A
16	6	M	1	A
17	7	M	1	A
18	3	M	1	A
19	0.3	M	1	A
20	1	F	5	A, A, A, A, A
21	0.8	M	1	A
22	6	M	1	A
23	12	M	3	A, A, A

M: male

F: female

Genotyping of environmental strains demonstrated that 4 isolates had different profiles (B, D, I) not corresponding to any of the profiles isolated from clinical specimens.

Table 2 illustrated the antimicrobial susceptibility profile of *P. aeruginosa*. Both environmental and clinical isolates were demonstrated to be susceptible to piperacillin-tazobactam. All clinical isolates were susceptible to ceftazidime and cefepime followed by ticarcillin, aztreonam, amikacin and tobramycin (96.5%). Trimethoprim-sulfamethoxazole was the least active antimicrobial agent against *P. aeruginosa* isolates (96.5% resistant). The highest percentage of resistance to gentamycin, tobramycin, aztreonam, ceftazidime, cefepime and ticarcillin was found in environmental isolates.

**Table 2:** In vitro antimicrobial susceptibility pattern of *P. aeruginosa* isolated from CF patients.

Antibiotic	Susceptibility of environmental isolates (%)	Susceptibility of clinical isolates (%)
peperacillin-tazobactam	100	100
ticarcillin	60	96.5
ceftazidime	60	100
cefepime	60	100
aztreonam	60	96.5
amikacin	100	96.5
tobramycin	60	96.5
gentamycin	30	82
ciprofloxacin	90	79
meropenem	100	93
imipenem	100	90
ertapenem	30	18
doripenem	100	90
Trimethoprim-sulfamethoxazole	0	3.5

## DISCUSSION

*P. aeruginosa* is a significant cause of nosocomial infection and the primary infectious cause of morbidity and mortality in CF patients (10). In our study, the majority of *P. aeruginosa* isolated from CF patients were related (89%), while a clonal strain which was previously described in cystic fibrosis units was 59% (11).

In our study, in majority of cases, multiple isolates of *P. aeruginosa* recovered from a patient were more likely to be related than unrelated. This shows the presence of same clonal types in the same patient.

Isolates of 3 patients displayed an A profile and other different patterns. This means that different genotypes may be recovered beside the consistent genotypes.

In our center, normally, CF patients are assigned to the hospital respiratory ward and are not segregated from other patients, but they might also be moved to other wards when the degree of care they require changes. In addition, patients may be transiently admitted to a ward that does not match the medical specialty of their illness when there is no bed available and an immediate admission is necessary. This recurrent patient movement may result in the transmission of bacteria between CF patients and other patients around the hospital especially if proper infection control is not established (12). Our study provides further support for segregation of *P. aeruginosa*-positive patients from other patients at least 3 ft apart to minimize respiratory droplet transmission although recent data suggests that droplets may travel as far as 6–10 ft (13). However, colonization with genotypically identical isolates has been documented for CF patients who were separated from each other, suggesting a possible transmission and acquisition from an environmental source (14). In this center, patient care equipment including suctions and nebulizers harbor related strains that may become further contaminated in the unit and might be inadequately cleaned, disinfected or sterilized. Therefore, transmission from environmental surfaces or by airborne route can occur in special circumstances such as intubated patients.

It may prove that both person-to-person transmission and acquisition from an environmental source have occurred concurrently. The current findings provide further evidence that these equipments should have single patient use and be cleaned and dried thoroughly after each use (15).

Our isolates show 100% susceptiblility to piperacillin-tazobactam, so these drugs can safely be used in patients with cystic fibrosis as an empiric treatment when resistance to common first-line antimicrobials has emerged (16).

In our study, not only colonized environmental surfaces can serve as a reservoir for *P. aeruginosa* but also resistance rates from these isolates are substantially higher than those found in isolates from patients. Therefore, strategies to reduce further spread are needed and priority should be given to improve of infection control practices in the hospital.

Nosocomial transmission can be prevented with simple hygienic measures and elimination of contaminated material. Transmission of *P. aeruginosa* from patient-to-patient can lead to spread of clonal strains in other wards of the hospital. Thus, future support for segregation of patients is necessary.
